# Effects of Dietary Zinc Cysteamine Supplementation on Growth Performance, Physiological Responses, and Fecal Microbiota in Weaned Foals

**DOI:** 10.3390/ani16101568

**Published:** 2026-05-21

**Authors:** Jie Ren, Chaoyu Ma, Kailun Yang, Xiaobin Li, Fan Yang, Xinsheng Guo, Xinkui Yao, Caidie Wang

**Affiliations:** College of Animal Science, Xinjiang Agricultural University, Urumqi 830052, China; 15133391369@163.com (J.R.); 13809951421@163.com (C.M.); m13934057294@163.com (K.Y.); lxb262819@163.com (X.L.); 15132311203@163.com (F.Y.); 13629439845@163.com (X.G.); 18723181520@163.com (X.Y.)

**Keywords:** zinc cysteamine, weaned foals, growth performance, antioxidant capacity, gut microbiota

## Abstract

Zn-CS chelate has shown beneficial effects on gut health and growth in piglets, but its impact on weaned foals remains largely unknown. This study evaluated the effects of dietary Zn-CS supplementation on growth performance, nutrient digestibility, physiological status, and fecal microbiota in weaned Ili foals. Thirty-two six-month-old foals were randomly assigned to four treatment groups receiving 0, 2, 4, or 6 mg Zn-CS/kg body weight per day for 90 days. Growth performance, nutrient digestibility, plasma biochemical parameters, liver function enzymes, serum hormones, antioxidant indices, fecal pH, volatile fatty acids, and fecal microbial composition were measured. Dietary Zn-CS supplementation significantly increased final body weight, total weight gain, and average daily gain (*p* < 0.05), while linearly improving body size indicators. Apparent digestibility of dry matter, digestible energy, metabolizable energy, and acid detergent fiber was markedly enhanced (*p* < 0.05). Zn-CS supplementation also effectively regulated plasma albumin and total cholesterol levels and hepatic enzyme activities, and strongly enhanced antioxidant function by increasing superoxide dismutase, glutathione peroxidase, catalase activities, and total antioxidant capacity, while reducing malondialdehyde content (*p* < 0.01). Additionally, Zn-CS upregulated plasma growth hormone, insulin, and triiodothyronine concentrations, decreased somatostatin secretion (*p* < 0.05), reduced fecal pH, and increased VFA contents. Notably, Zn-CS reshaped the fecal microbial structure by increasing beneficial bacteria and inhibiting potential pathogens. In conclusion, dietary Zn-CS supplementation effectively promotes growth and health in weaned Ili foals, with 6 mg/kg BW/day being the optimal supplemental dose under experimental conditions.

## Simple Summary

Weaning is a critical stage in foal development and can be associated with nutritional stress, impaired growth, and intestinal imbalance. Zinc cysteamine (Zn-CS), a functional feed additive, has been shown to enhance antioxidant capacity, immune function, and growth performance in livestock. In this study, Zn-CS supplementation improved nutrient digestibility, growth performance, antioxidant status, and endocrine regulation in weaned foals. Additionally, Zn-CS modulated gut microbial composition and reduced fecal pH. These findings suggest that Zn-CS is a promising nutritional strategy to support the health and development of foals during the weaning period.

## 1. Introduction

Weaning represents a critical stage in foal development and may be associated with impaired growth and intestinal instability [1]. Following weaning, the digestive system of foals undergoes a transitional adaptation to solid feed, accompanied by modifications in the intestinal microbiota. These changes may predispose foals to transient digestive disturbances, including episodes of diarrhea [2]. Additionally, weaning may elicit measurable stress responses. The reduction in antioxidant intake—particularly vitamins E and C—previously supplied through maternal milk can lead to increased production of reactive oxygen species, contributing to oxidative stress, cellular damage, and the initiation of inflammatory processes [3,4,5].

Zn-CS, a cysteamine (CS)-chelated form of zinc (ZN), is a novel feed additive noted for its chemical stability. It is synthesized by chelating CS with Zn ions (Zn^2+^) derived from zinc sulfate. Zn^2+^ also promotes the synthesis of metallothionein, a cysteine-rich protein with a high capacity for scavenging hydroxyl radicals. Studies have demonstrated that Zn, through binding to cysteine residues, plays critical roles in regulating protein structure, enzyme activity, and cellular signaling pathways [6]. Zhu et al. reported that supplementation with Zn-CS chelate improved growth performance, serum biochemical indices, nutrient digestibility, and fecal microbial composition in piglets [7]. Similarly, Wang et al. found that Zn-CS chelate positively affected growth performance, apparent nutrient digestibility, carcass traits, and meat quality in finishing pigs [8]. A systematic review by Welk et al. highlighted that different weaning strategies significantly influenced growth, behavior, and health in dairy calves [9]. Meng et al. further demonstrated that weaning stress can reshape gut microbiota and alter the expression of intestinal genes involved in nutrient metabolism in pigs [10]. Moreover, Yoon et al. indicated that *Lactiplantibacillus argentoratensis* AGMB00912 alleviated diarrhea and promoted growth performance in piglets during the weaning transition [11]. To date, no studies have examined the effects of Zn-CS on equine digestive performance, immune function, nutrient digestibility, or other health parameters. Implementing effective nutritional strategies during weaning is therefore crucial to support foal development and optimize performance. This study represents the first investigation of Zn-CS supplementation in weaned foals, assessing its effects on growth performance, antioxidant capacity, immune status, and nutrient digestibility, and providing a potential nutritional strategy to enhance health and productivity during the post-weaning period.

## 2. Materials and Methods

### 2.1. Study Area

The experiment was conducted at a military horse farm in Zhaosu County, Yili Kazakh Autonomous Prefecture, Xinjiang, China (43°55′19.54″ N, 81°19′38.87″ E) from November 2023 to February 2024. All experimental procedures were approved by the Animal Welfare Ethics Committee of Xinjiang Agricultural University (Approval No. 2022020).

### 2.2. Animals and Experimental Design

A total of 32 healthy weaned Ili (Yili) horses (6 months old; average body weight 156.20 ± 11.16 kg; equal numbers of males and females) were selected for the study. The foals were randomly assigned to four groups (*n* = 8 per group): the study comprised one control group and three treatment groups: group I, group II, and group III, which were orally administered Zn-CS at doses of 2, 4, and 6 mg·kg^−1^·d^−1^, respectively. The experiment was conducted over 90 days following a 14-day adaptation period immediately after weaning. During this period, all foals were managed under identical feeding and husbandry conditions. The supplementation levels were selected based on previous studies [7].

The trial was performed under a semi-intensive equine management system, including stable housing, regular feeding, and daily outdoor grazing. The control group received no Zn-CS supplementation, while groups I, II, and III were provided the designated Zn-CS doses once daily. Concentrate feed was offered at 0.0065 kg/kg body weight/day (based on NRC 2007) [12], divided into two equal portions and administered at 09:30 and 17:30. Zn-CS was accurately weighed using an analytical balance and mixed thoroughly into the morning feed to ensure complete intake by each foal. Foals were allowed free movement in the exercise area between 10:00 and 17:00, and were subsequently maintained under group housing conditions in outdoor pens from 18:00 to 09:00. To prevent competition and mixed consumption, foals were individually separated in independent stalls during feeding. Each foal received its daily ration in a separate feeding bucket. Feed offered to each foal was weighed precisely before feeding, and any residual or spilled feed was collected and weighed. The actual daily feed intake was calculated by subtracting the residual and spilled feed from the initial supplied weight. Routine management included daily cleaning of the barn, manure removal, and environmental disinfection. Health monitoring, physical examinations, parasite prevention, and routine immunizations were conducted in accordance with farm regulations. No additional medical interventions were applied during the experimental period. The composition and nutritional content of the concentrate supplement are presented in Table 1.

#### 2.2.1. Growth Performance Measurement

Foal body weight was recorded on days 0, 30, 60, and 90 prior to the morning feeding using a digital livestock scale (Tru-Test ID 3000; accuracy 0.5 kg; range 0–1000 kg; Ili Horse Research and Breeding Center, Xinjiang Zhaosu Xiyu Horse Industry Co., Ltd., Xinjiang, China). Body measurements, including body height, body length, chest circumference, and cannon circumference, were obtained at the start and end of the experiment by the same trained personnel using standard measuring tools in accordance with conventional equine measurement standards. During measurements, foals were maintained in a natural upright standing posture to ensure accuracy and consistency. These indices reflect skeletal growth, body size, and overall physical development, serving as important indicators of growth performance in weaned foals.

#### 2.2.2. Determination of Plasma Biochemical Parameters

Plasma biochemical parameters related to nitrogen metabolism, glucose and lipid metabolism, enzyme activity, and mineral ions were determined using an automatic biochemical analyzer (BS-240VET, Mindray Bio-Medical Electronics Co., Ltd., Shenzhen, China). Nitrogen metabolism indicators included total protein (TP), albumin (ALB), globulin (GLB), and urea nitrogen (UN). Glucose and lipid metabolism indicators comprised glucose (Glu), total bilirubin (T-Bil), serum creatinine (CREA-S), indirect bilirubin (I-Bil), triglycerides (TG), and total cholesterol (TC). Enzyme activity indices included alanine aminotransferase (ALT), aspartate aminotransferase (AST), alkaline phosphatase (ALP), γ-glutamyl transferase (γ-GT), creatine kinase (CK), and lactate dehydrogenase (LDH). Mineral ions measured were calcium (Ca^2+^), inorganic phosphorus (P), and magnesium (Mg^2+^). All indices were analyzed according to standard biochemical procedures, using commercial assay kits supplied by Beijing Huaying Biotechnology Research Institute (Beijing, China).

#### 2.2.3. Determination of Plasma Antioxidant Index

Antioxidant capacity was evaluated by measuring total antioxidant capacity (T-AOC), superoxide dismutase (SOD), glutathione peroxidase (GSH-Px), catalase (CAT), and malondialdehyde (MDA). T-AOC was measured using the ABTS method, SOD activity was determined using the xanthine oxidase method, GSH-Px and CAT activities were measured using colorimetric assays, and MDA content was determined by the thiobarbituric acid (TBA) method. All plasma samples were pretreated by refrigerated centrifugation prior to analysis to prevent oxidative degradation. Antioxidant indices were quantified using commercial kits from Beijing Huaying Biotechnology Research Institute (Beijing, China), following the manufacturer’s instructions.

#### 2.2.4. Determination of Plasma Growth Hormones

Plasma hormone levels were measured using commercial ELISA kits obtained from Beijing Huaying Biotechnology Research Institute, strictly adhering to the manufacturer’s standard protocols. Hormonal indices included growth hormone (GH), insulin (INS), thyroxine (T_4_), triiodothyronine (T_3_), and somatostatin (SS), all quantified using double-antibody sandwich ELISA methodology.

#### 2.2.5. Blood and Fecal Sample Collection

Blood samples were collected from the jugular vein of each foal on days 0, 30, 60, and 90, prior to morning feeding and body weight measurement. Samples were drawn into two 5 mL heparinized tubes per foal and centrifuged at 3500 rpm for 15 min to separate plasma. The plasma was aliquoted into 2 mL Eppendorf tubes, labeled, and stored at −80 °C for subsequent analysis of plasma biochemical parameters, hormone levels, and antioxidant indices. Fresh fecal samples were collected from each foal on the final day of the experiment using disposable sterile gloves. Samples were obtained directly from the rectum rather than from the ground to avoid contamination. Approximately 50 g of feces per collection was placed into sterile RNA-free cryogenic tubes. Three samples were collected per foal, immediately frozen in liquid nitrogen, and then stored at −80 °C for subsequent analysis of fecal microbial diversity, volatile fatty acids (VFAs), and pH. Immediate freezing in liquid nitrogen rapidly inhibits intracellular enzyme activity and microbial metabolism, thereby preserving biomolecules, including DNA, RNA, proteins, and metabolites, and preventing alterations in biochemical and microbiological indicators.

#### 2.2.6. Sample Collection for Nutrient Digestibility and Metabolism

##### Feed Sample Collection

A 6-day digestibility trial was conducted from day 85 to day 90 of the experimental period. During this trial, foals were housed individually indoors under consistent environmental conditions. Daily procedures were performed in a strict chronological order. Before the morning feeding at 09:00, the amount of feed offered to each foal was accurately weighed. Approximately 100 g of the daily fresh diet was collected for routine nutrient composition analysis and stored in sealed bags. Representative total feed samples (500 g) were collected using the five-point sampling method throughout the digestibility trial to determine nutrient intake accurately. Residual feed was completely collected and weighed prior to the next morning feeding to calculate actual daily dry matter (DM) intake. In this study, any residual or spilled feed was collected and weighed as described in Section 2.2. Collected feed samples were then air-dried, reweighed, ground through a sieve, and stored for subsequent nutrient analysis.

##### Fecal and Urine Sample Collection

During the digestibility trial, total fecal collection was performed for each foal. All daily feces were collected into containers containing 10% dilute sulfuric acid to fix nitrogen. The daily total fecal weight per foal was recorded. Fresh feces were thoroughly homogenized manually, and a 10% subsample of the daily feces was collected via the quartering method from the upper, middle, and lower portions of the mixture and stored at −20 °C. After the 6-day collection period, all daily fecal samples per foal were combined, mixed uniformly, air-dried, and reweighed. Approximately 1 kg of the composite fecal sample per foal was retained at room temperature for subsequent analysis.

Daily urine output from each foal was collected into containers pre-filled with 5% (*v*/*v*) dilute sulfuric acid to acidify the samples, thereby reducing ammonia volatilization and preventing nitrogen loss. Total daily urine volume was recorded, and all urine samples were stored at −20 °C. After the 6-day trial, daily urine samples per foal were pooled, mixed thoroughly, and stored for subsequent nutrient determination.

#### 2.2.7. Determination of Nutrient Compositions

Feed, concentrate supplements, and fecal samples were ground using a high-speed grinder and passed through a 40-mesh sieve prior to analysis. Gross energy (GE) was determined using an automatic calorimeter (OR2014, Shanghai, China). Nutrient composition—including DM, organic matter (OM), GE, crude protein (CP), neutral detergent fiber (NDF), acid detergent fiber (ADF), Ca, and P—was analyzed according to the national standards of the People’s Republic of China (GB/T series) [13], relevant agricultural industry standards (NY/T series) [14], and internationally recognized AOAC official methods [15].

#### 2.2.8. Determination of Fecal pH and Volatile Fatty Acids

##### Determination of Fecal pH

For fecal pH measurement, samples were thoroughly mixed, and 10 g of feces was diluted 1:1 (*w*/*v*) with distilled water. The mixture was stirred at room temperature for 3–5 min, and pH was measured using a calibrated portable pH meter with 0.01 accuracy (FiveEasy22-Meter, Mettler-Toledo, Shanghai, China).

##### Determination of Volatile Fatty Acids

VFAs were quantified using a gas chromatograph (GC-2010, Shimadzu, Kyoto, Japan) equipped with a Stabilwax capillary column. 4-Methylvaleric acid served as the internal standard. The chromatographic analysis was performed under the following conditions: the injector temperature was set at 230 °C; the initial column temperature was maintained at 55 °C, then increased at a rate of 13 °C/min to 200 °C and held for 0.5 min; the flame ionization detector (FID) temperature was set at 240 °C.

For sample preparation, 1 mL of filtrate was centrifuged at 15,000 rpm for 5 min, and 0.5 mL of the supernatant was transferred to a 1.5 mL centrifuge tube. Subsequently, 0.5 mL of 10% trichloroacetic acid (TCA) and 0.1 mL of 40 mmol/L 4-methylvaleric acid (internal standard) were added. The mixture was vortexed, incubated for 20 min, and centrifuged at 20,000× *g* for 15 min. A 1.0 mL aliquot of the supernatant was transferred into a sample vial, and 0.5 μL was injected for GC analysis. VFA concentrations were calculated using the internal standard method, establishing a regression equation based on the ratio of VFA concentration (*y*-axis) to peak height ratio (*x*-axis).

Reagents were prepared as follows: a 10% (*w*/*v*) TCA solution was made by dissolving 10 g of TCA in 100 mL of distilled water. A 40 mmol·L^–1^ 4-methylvaleric acid solution was prepared by diluting 50.2 μL of the acid to 10 mL with distilled water. The standard curve for VFA determination exhibited excellent linearity (R^2^ > 0.999), as presented in Table 2.

### 2.3. Statistical Analysis

Data analysis was performed using SPSS 27.0 software (IBM Corp., Armonk, NY, USA). Repeatedly measured variables—including growth performance, plasma biochemical parameters, antioxidant indices, and hormone levels—were analyzed using two-way ANOVA, with dietary Zn-CS supplementation level and sampling time as the main effects, as well as their interaction. When significant differences were detected, Duncan’s multiple range test was applied for post hoc comparisons.

Variables measured only once at the end of the experiment, such as apparent nutrient digestibility, fecal pH, VFA concentrations, and fecal microbial diversity indices, were analyzed using one-way ANOVA. Linear and quadratic effects of increasing Zn-CS supplementation levels were further evaluated via orthogonal polynomial contrasts. Microbial beta diversity was assessed based on UniFrac distances using QIIME2 (version 2023.2), and principal component analysis (PCA) and principal coordinates analysis (PCoA) were performed in R software (4.2.1). Differentially abundant taxa were identified using LEfSe (1.1) analysis with a linear discriminant analysis (LDA) score threshold of 4. Pearson correlation analysis was employed to evaluate associations between the top 30 genera and nutrient digestibility, hormone levels, fecal pH, and VFA concentrations. All results are expressed as mean ± SD. Statistical significance was defined as *p* < 0.05, and high significance as *p* < 0.01.

## 3. Results

### 3.1. Effects of Zn-CS on Apparent Nutrient Digestibility in Weaned Foals

The effects of dietary Zn-CS supplementation on apparent nutrient digestibility and nutrient metabolism in weaned foals are summarized in Table 3. Zn-CS supplementation significantly increased DM, digestible energy (DE), and metabolizable energy (ME) compared with the control group (*p* < 0.05), showing a linear response to increasing supplementation levels. ADF digestibility was also significantly enhanced (*p* < 0.05), whereas no significant differences were observed for CP, NDF, Ca, or P (*p* > 0.05).

### 3.2. Effects of Zn-CS on Growth Performance of Weaned Foals

**Figure 1:**
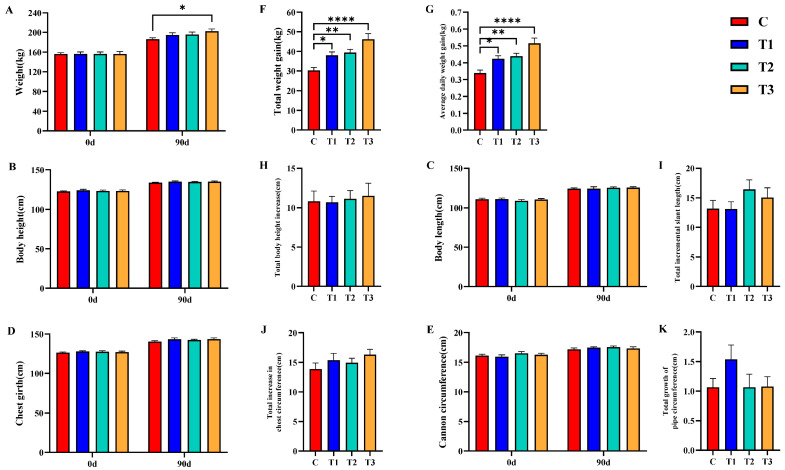
Effects of dietary Zn-CS supplementation on growth performance parameters of weaned foals over the 90-day experimental period. Note: (**A**) body weight; (**B**) body height; (**C**) body length; (**D**) chest circumference; (**E**) cannon circumference; (**F**) total weight gain; (**G**) average daily gain; (**H**) total height gain; (**I**) total length gain; (**J**) total chest circumference gain; (**K**) Total growth of pipe circumference. * indicates a significant difference at *p* < 0.05, ** represents an extremely significant difference at *p* < 0.01, and **** indicates an ultra-significant difference at *p* < 0.0001. (C: Control group; T1: 2 mg/kg BW/d; T2: 4 mg/kg BW/d; T3: 6 mg/kg BW/d).

Overall, dietary Zn-CS supplementation improved growth performance in weaned foals, particularly with respect to body weight gain and average daily gain (ADG). Statistical analysis indicated significant effects of dietary Zn-CS supplementation, sampling time, and their interaction on growth performance (Figure 1). Compared with the control group, Zn-CS supplementation increased body weight, total weight gain, and ADG throughout the experimental period, with the greatest improvement generally observed in the 6 mg/kg group (*p* < 0.05). These variations in growth-related parameters may reflect the typical developmental trajectory of foals throughout the 90-day trial period.

### 3.3. Effects of Zinc Cysteamine on Plasma Biochemical Indexes of Weaned Foals

Analysis of plasma biochemical parameters revealed significant differential effects of dietary Zn-CS supplementation, sampling time, and their interaction (Table 4). Dietary treatment significantly influenced ALB, CREA-S, TC, T-bil-D, AST, ALT, ALP, and Mg concentrations (*p* < 0.05), whereas TP, GLB, UN, Glu, γ-GT, CK, AST/ALT ratio, Ca, and P were not significantly affected by treatment (*p* > 0.05). Sampling time significantly affected most biochemical parameters, including TP, ALB, GLB, UREA, CREA-S, Glu-G, TC, T-bil-D, AST, ALT, ALP, CK, AST/ALT, and Ca (*p* < 0.05). A significant treatment × time interaction was observed for TP and UREA (*p* < 0.05), whereas no significant interactions were detected for the remaining parameters (*p* > 0.05). Compared with the control group, Zn-CS supplementation increased ALB, TC, AST, ALT, and ALP concentrations at specific sampling points, while CREA-S and T-bil-D were also altered, and Mg concentrations were reduced in some supplemented groups.

### 3.4. Effects of Zinc Cysteamine on Plasma Hormones in Weaned Foals

**Figure 2:**
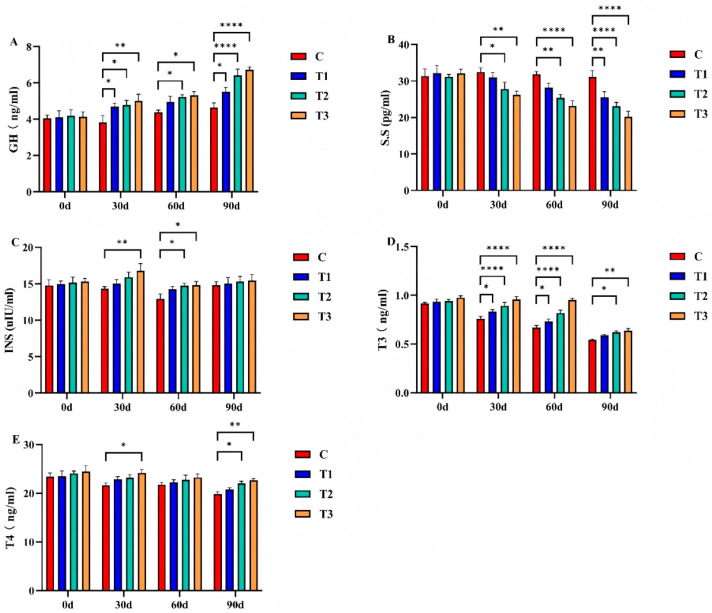
Effects of zinc cysteamine on plasma hormones in weaned foals. Note: (**A**): growth hormone; (**B**): somatostatin; (**C**): insulin; (**D**): triiodothyronine; (**E**): thyroxine. * indicates a significant difference at *p* < 0.05, ** represents an extremely significant difference at *p* < 0.01, and **** indicates an ultra-significant difference at *p* < 0.0001. (C: Control group; T1: 2 mg/kg BW/d; T2: 4 mg/kg BW/d; T3: 6 mg/kg BW/d). GH, growth hormone; S.S, somatostatin; INS, insulin; T_3_, triiodothyronine; T_4_, thyroxine.

Dietary Zn-CS supplementation also influenced plasma hormone levels in weaned foals (Figure 2). Compared with the control group, plasma concentrations of GH, INS, and T_3_ were significantly increased in Zn-CS-supplemented groups (*p* < 0.05), whereas SS levels were significantly decreased (*p* < 0.05). T_4_ concentrations exhibited an increasing trend in the supplemented groups, with significant differences observed at specific sampling times. Hormone levels fluctuated dynamically throughout the experimental period, and the magnitude of these changes varied among treatment groups. In general, the regulatory effects of Zn-CS supplementation on endocrine indices were more pronounced at later sampling points, particularly in foals receiving higher supplementation levels.

### 3.5. Effects of Zinc Cysteamine on Plasma Antioxidation of Weaned Foals

Plasma antioxidant indices were affected by both dietary Zn-CS supplementation and sampling time (Table 5). Compared with the control group, Zn-CS supplementation significantly increased the activities of SOD, GSH-Px, T-AOC, and CAT (*p* < 0.01), while MDA concentration was significantly reduced (*p* < 0.01). Sampling time also had a significant effect on all antioxidant parameters (*p* < 0.01), whereas no significant treatment × time interactions were observed for these indices (*p* > 0.05). Throughout the experimental period, foals receiving Zn-CS consistently exhibited higher antioxidant enzyme activities and lower lipid peroxidation levels compared with the control group, and these effects generally increased with higher supplementation levels.

### 3.6. Effects of Zinc Cysteamine on pH and VFA of Feces of Weaned Foals

Dietary Zn-CS supplementation significantly decreased fecal pH in a dose-dependent manner (*p* < 0.05; Table 6). For most individual volatile fatty acids (VFAs), no significant differences were detected among treatment groups in pairwise comparisons. However, a significant linear effect of dietary Zn-CS supplementation was observed for valerate concentration (*p* = 0.035), indicating a dose-dependent increase with increasing Zn-CS levels. No significant linear or quadratic trends were found for the other VFAs (*p* > 0.05).

### 3.7. Effects of Zinc Cysteamine on the Diversity of Fecal Flora of Weaned Foals

The effect of zinc cysteamine on the diversity of fecal microbiota in weaned foals is presented in Table 7. As shown in this table, Dietary Zn-CS supplementation did not significantly affect alpha diversity indices (Shannon, Simpson, Chao1, and observed species; *p* > 0.05). Moreover, orthogonal polynomial contrast analysis revealed no significant linear or quadratic trends for any of these indices with increasing Zn-CS levels (*p* > 0.05 for all).

### 3.8. Effects of Supplementing Zn-CS on the Fecal Flora of Weaned Foals

**Figure 3:**
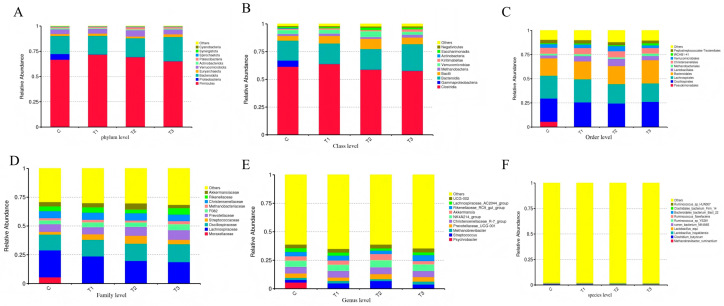
Effects of Zn-CS supplementation on fecal flora diversity at different taxonomic levels (phylum to species). Note: Relative abundance results of bacteria, (**A**): Phylum level; (**B**): Class level; (**C**): Order level; (**D**): Family level; (**E**): Genus level; (**F**): Species level. (C: Control group; T1: 2 mg/kg BW/d; T2: 4 mg/kg BW/d; T3: 6 mg/kg BW/d).

**Figure 4:**
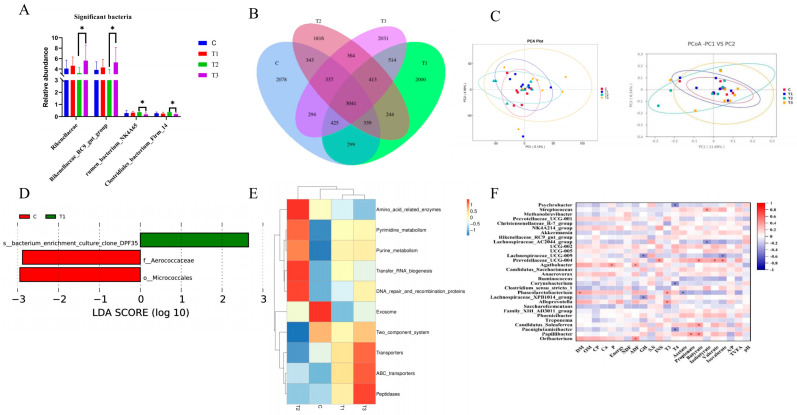
Effects of Zn-CS supplementation on fecal microbiota composition, functional potential, and correlations. Note: (**A**): Significant bacteria; (**B**): Venn; (**C**): Principal component analysis; (**D**): LEfSe; (**E**): Prediction of Tax4Fun Function; (**F**): Correlation analysis. * indicates a significant difference at *p* < 0.05. (C: Control group; T1: 2 mg/kg BW/d; T2: 4 mg/kg BW/d; T3: 6 mg/kg BW/d).

At the phylum level, Firmicutes, Proteobacteria, and Bacteroidota were the dominant bacterial groups across all treatments (Figure 3). Although Zn-CS supplementation did not significantly alter the overall microbial structure, it induced shifts in the relative abundance of specific taxa. At the genus level, the abundance of *Streptococcus* in each experimental group was higher than that in the control group, but there was no significant difference (*p* > 0.05). The abundance of *Rikenellaceae_RC9_gut_group* in group III was significantly higher than that in group II (*p* < 0.05). Beta diversity analysis (Figure 4) indicated partial separation among groups, principal coordinate analysis (PCoA) based on weighted UniFrac distances suggested a trend of partial separation among groups, Pairwise PERMANOVA (Adonis) analysis confirmed partial separation of microbial community structure among groups, with R^2^ values ranging from 0.055 to 0.079, indicating weak-to-moderate community differences. Detailed pairwise results are provided in Supplementary Table S7. LEfSe analysis further identified several taxa with differential abundance between groups, with specific enrichment observed in both control and treatment groups.

According to the correlation analysis, regarding nutrient digestibility: *Phascolarctobacterium* was significantly positively correlated with dry matter digestibility; *Agathobacter* was significantly positively correlated with phosphorus digestibility; and both *Agathobacter* and *Oribacterium* were significantly positively correlated with ADF digestibility. Regarding growth hormones: *Lachnospiraceae UCG-009* and *Lachnospiraceae XPB1014* group were significantly negatively correlated with GH; *Prevotellaceae UCG-004* was significantly positively correlated with INS; *Phascolarctobacterium* and *Alloprevotella* were significantly positively correlated with T_3_; while *Psychrobacter*, *Corynebacterium*, and *Glutamicibacter* were significantly negatively correlated with T_4_. Regarding volatile fatty acids: *Phascolarctobacterium* was significantly negatively correlated with acetic acid; *Papillibacter* was significantly positively correlated with propionic acid; *Prevotellaceae UCG-004*, *Candidatus Saccharimonas*, and *Papillibacter* were significantly positively correlated with butyric acid; *Streptococcus* was significantly positively correlated with isobutyric acid, whereas the *Lachnospiraceae AC2044* group was significantly negatively correlated with isobutyric acid; *Prevotellaceae UCG-004* was significantly positively correlated with valeric acid; and for isovaleric acid, *Lachnospiraceae UCG-009* showed a significant negative correlation, while *Prevotellaceae UCG-004* showed a significant positive correlation.

## 4. Discussion

Weight and body size are key indicators for evaluating foal growth and development, directly reflecting overall health, dietary nutrition, and bone/muscle development [16,17]. The period from weaning to one year of age exerts the greatest impact on foal growth and development. During this period, increases in body weight and morphometric parameters occur at a slower rate compared to suckling foals. This reduced growth performance can be attributed to dietary transitions, weaning-induced stress, and the immaturity of the intestinal microbiota [18]. Studies have shown that Zn-CS has positive effects on livestock and poultry, such as promoting the secretion of growth hormone, improving the absorption efficiency of nutrients and antioxidation [19]. Wang et al. showed that adding Zn-CS to the diets of fattening pigs in the test group can not only significantly increase the average daily gain, but also reduce the feed-to-weight ratio compared with the control group [8]. Dietary Zn-CS supplementation has been reported to improve growth performance, reduce diarrhea and intestinal *Escherichia coli* in piglets [20]. This study showed that Zn-CS supplementation significantly increased weaned foal weight, with a tendency of increased body size, consistent with Zhang’s research [20]. This may be related to Zn-CS promoting digestive enzyme secretion and activity to improve nutrient digestibility, as well as reducing intestinal epithelial cell damage and protecting intestinal mucosal integrity to enhance nutrient absorption efficiency [21]; In addition, these effects may also be associated with the antioxidant properties of Zn-CS. It has been reported that weaning stress in foals may increase the production of free radicals, leading to a reduction in the body’s antioxidant capacity [22]. In contrast, Zn-CS contains sulfhydryl groups (-SH) that can donate electrons to free radicals, directly neutralizing them. It also provides zinc ions, which may enhance the activity of antioxidant enzymes such as SOD and GSH-Px. SOD converts superoxide radicals into hydrogen peroxide and oxygen. GSH-Px then reduces peroxides, including hydrogen peroxide, thereby limiting lipid peroxidation in vivo. This synergistic effect of cysteine and zinc ions reduces most free radicals in the body to water and alcohol, thus reducing the damage of oxidative stress to the body more effectively [23].

Apparent nutrient digestibility reflects the efficiency of nutrient utilization and energy deposition in animal tissues. Dietary Zn-CS supplementation significantly improves DM, CP, and Ca digestibility, increases average daily gain, and reduces feed conversion ratio in piglets [7]. In finishing sheep, Zn-CS supplementation enhanced the apparent digestibility of DM, OM, and ether extract (EE), as well as feed utilization efficiency [24,25]. In the current study, dietary Zn-CS supplementation significantly increased DM, DE, ME, and ADF digestibility in a linear dose-dependent manner in weaned foals, whereas no significant differences were observed for CP, NDF, Ca, or P digestibility. To explore potential mechanisms, Pearson correlation analysis was performed between the top 30 intestinal bacterial genera and nutrient digestibility. Results showed that *Agathobacterium* abundance was positively correlated with P and ADF digestibility. In monogastric herbivores like foals, energy is largely derived from hindgut fermentation of fiber to VFAs. *Agathobacterium* collectively promote fiber degradation and energy supply [26]. Collectively, these results suggest that dietary Zn-CS improves intestinal microbial community structure and further enhances nutrient digestibility in weaned foals.

Plasma nitrogen metabolism parameters directly reflect the digestion, absorption, and utilization of dietary protein, and mainly include TP, ALB, GLB, UN, and CREA-S [27,28,29]. Under consistent dietary protein intake, elevated serum UN generally indicates reduced protein utilization efficiency and may also reflect inflammatory responses or impaired renal function [30]. Creatinine is primarily produced from muscle creatine phosphate metabolism, and its concentration is positively correlated with muscle mass. Increased creatinine levels may indicate reduced renal filtration capacity or enhanced muscle deposition, whereas decreased levels can result from insufficient protein intake, malnutrition, or muscle atrophy [31]. Previous studies reported that dietary Zn-CS supplementation (3, 5, and 7 mg/kg BW) significantly increased TP and decreased UN in foals [32]. In contrast, Ma et al. found that Zn-CS did not significantly affect plasma nitrogen metabolism in suckling foals, with levels rising until day 60 and stabilizing by day 90 [33]. In the present study, dietary Zn-CS significantly affected ALB, CREA-S, TC, T-Bil-D, AST, ALT, ALP, and Mg concentrations, likely reflecting Zn-CS’s ability to enhance protein synthesis and reduce protein catabolism, thereby improving nitrogen metabolism.

Dietary carbohydrates, particularly non-structural forms, are hydrolyzed into glucose by amylase in the stomach and small intestine, providing the body’s primary energy source [34,35]. Abnormal elevations in TC may indicate impaired liver or endocrine function, whereas decreases may reflect inadequate nutrient intake or thyroid dysfunction [36,37,38]. After weaning, T-Bil levels in foals may fluctuate due to dietary changes, reduced nutrient digestibility, and altered metabolic homeostasis. Key plasma enzyme markers, including AST, ALT, ALP, γ-GT, CK, and LDH, provide insights into liver and muscle health. CK is predominantly found in muscle tissue and functions in energy metabolism, providing a measure of muscle tissue and skeletal muscle development [39]. LDH primarily participates in glycolysis and the tricarboxylic acid cycle, catalyzing the conversion of lactic acid to pyruvate, and, together with the aforementioned transferases, serves as an important indicator for evaluating liver damage [40]. Liu et al. reported that increasing cysteamine hydrochloride supplementation (3, 15, and 30 g/kg) elevated ALT, AST, ALP, and CK activities in dairy cows, suggesting that Zn-CS compounds can modulate hepatic enzyme activity [41]. Similarly, in the present study, Zn-CS supplementation altered plasma enzyme indices in weaned foals, consistent with enhanced energy and protein metabolism.

Oxidative stress disrupts the balance between oxidants and antioxidants, impairing growth [42,43]. Zn is an essential cofactor for antioxidant enzymes, while CS serves as a glutathione precursor; together, they synergistically scavenge reactive oxygen species and reduce oxidative damage [44,45]. Previous studies have shown that CS and Zn-CS improve antioxidant capacity and decrease MDA levels in pigs and sows [46,47]. Excessive oxidative stress induces lipid peroxidation and MDA accumulation, which are counteracted by antioxidant enzymes such as SOD, GSH-Px, and glutathione [48]. In this study, dietary Zn-CS significantly increased plasma SOD, GSH-Px, T-AOC, and CAT activities, while reducing MDA concentration in weaned foals. This effect is likely mediated by enhanced glutathione synthesis and free radical scavenging, along with taurine derived from CS mitigating oxidative injury [49]. Collectively, these findings indicate that dietary Zn-CS effectively alleviates oxidative stress and supports healthy growth in weaned foals.

Zn-CS has been reported to positively influence animal growth and development, particularly by modulating growth-related hormone secretion [50]. CS promotes the release of GH and insulin-like growth factor 1 (IGF-1) by regulating the hypothalamic-pituitary axis and inhibiting SS [51]. Previous studies demonstrated that CS administration significantly elevated plasma GH, T_3_, and T_4_ in mice [52], enhanced GH pulsatile secretion in Hu sheep [53], and increased GH, INS, T_3_, and T_4_ while reducing SS in weaned piglets [54]. In the present study, dietary Zn-CS supplementation significantly increased plasma GH, INS, and T_3_ levels and decreased SS concentration compared with the control group. Since weaning stress typically causes a transient decline in GH secretion in foals, the elevated hormone levels observed in Zn-CS-supplemented foals suggest that Zn-CS can alleviate weaning stress and promote growth by modulating neuroendocrine hormone secretion.

GH secretion in foals appears closely linked to intestinal microbiota. Correlation analysis revealed that *Chaetomium* UCG-*009* and XPB1014 were negatively correlated with GH; *Prevotella* (*UCG-004*) was positively correlated with INS; *Koala* and *IsoPrevotella* were positively correlated with T_3_; while psychrophilic bacteria, *Corynebacterium*, and glutamate-associated bacteria were negatively correlated with T_4_. As key intestinal polysaccharide-degrading bacteria, *Prevotella* can break down cellulose and hemicellulose to produce short-chain fatty acids (SCFAs) [55]. *Koala* and *IsoPrevotella* may enhance T_3_ levels and energy metabolism via SCFA production [56]. By contrast, halophilic bacilli, Corynebacterium, and glutamate-type bacteria may suppress T_4_ secretion by inducing inflammation and intestinal dysbiosis, thereby impairing thyroid function. Collectively, these intestinal microbes participate in host energy metabolism, with SCFAs serving as energy substrates that modulate hormone secretion through effects on energy utilization and glucose homeostasis [57]. These results confirm that intestinal microbiota can exert regulatory effects on endocrine hormone secretion in foals. Fecal pH reflects the intestinal environment, microbial fermentation activity, and overall gut health, with a normal range of 6.0–7.5 [58]. VFAs, the main products of fiber fermentation, provide energy for intestinal epithelial cells and help maintain acid-base balance [59]. Previous studies have shown that CS-based additives can reduce fecal pH and enhance intestinal VFA concentrations by optimizing microbial fermentation [24,60]. In this study, Zn-CS supplementation significantly decreased fecal pH in weaned foals and slightly increased total and individual VFAs, although differences were not statistically significant. These improvements likely result from the combined antibacterial effects of Zn and CS, which inhibit pathogenic microbes, reduce ammonia production from protein fermentation, and promote beneficial bacteria to produce VFAs. Correlation analysis further indicated that Koala abundance was negatively associated with acetic acid, whereas *Prevotella UCG-004* and other genera were positively associated with propionic and butyric acid. In summary, dietary Zn-CS improved the intestinal fermentation environment by lowering fecal pH and promoting VFA synthesis.

Fecal bacterial diversity is an important indicator of intestinal microecological health and is closely related to nutrient digestion and growth performance in foals. Weaning-induced dietary changes substantially reshape the intestinal microbiota, with cellulolytic bacteria such as Ruminococcaceae and Lachnospiraceae dominating the hindgut. In this study, dietary Zn-CS supplementation tended to shift the dominant phyla in weaned foals toward Firmicutes and Bacteroidota. Consistent with previous research, Zn-CS numerically increased alpha diversity indices such as Chao1 and Shannon, with higher microbial richness in group I and fewer unique operational taxonomic units (OTUs) in the supplemented groups. Although Zn-CS did not significantly alter overall microbial alpha diversity, it moderately enriched beneficial bacteria and suppressed pathogens. Beta diversity analysis further indicated that Zn-CS supplementation, along with weaning stress and dietary transition, jointly influenced intestinal microbial community composition.

In this study, 16S rRNA high-throughput sequencing, LEfSe analysis, and Tax4Fun functional prediction were used to investigate intestinal microbial composition and function in weaned foals. Previous studies have demonstrated that Zn-CS can modulate intestinal microbiota in pigs and cashmere goats [24,47]. In our experiment, Zn-CS supplementation significantly altered the dominant bacterial community. All treatment groups showed increased relative abundances of Firmicutes and decreased Proteobacteria, accompanied by lower abundances of Clostridia, γ-proteobacteria, Pseudomonadales, Moraxellaceae, and psychrophilic bacilli. Notably, group II exhibited significantly higher levels of *rumen_bacterium_NK4A65* and *clostridials_bacterium_firm_14* compared with group III. These findings indicate that Zn-CS promotes beneficial Firmicutes while inhibiting potentially harmful Proteobacteria, thereby contributing to improved intestinal health in foals. LEfSe analysis revealed that *Micrococcus* and *Aerococcus* were enriched in the control group, whereas *Clone_DPF35* was enriched in group I. Functional prediction using Tax4Fun highlighted distinct metabolic profiles among groups: exosome and two-component system pathways were predominant in the control group; two-component systems, transporters, and peptidases were enriched in groups I and III; and amino acid metabolism, nucleic acid metabolism, DNA repair, and protein biosynthesis were enhanced in group II. *Micrococcus* and *Aerococcus* may support intestinal homeostasis through exosome-mediated regulation, whereas *Clone_DPF35* likely influences community structure via competitive colonization. The enhanced metabolic functions observed in the treatment groups align with the known biological effects of Zn-CS [61].

Overall, Zn-CS supplementation was associated with improved growth performance, enhanced antioxidant capacity, and modulation of selected physiological and microbial parameters in weaned foals. However, these conclusions are primarily based on associative analyses, and further studies are needed to clarify the underlying mechanisms.

## 5. Conclusions

In conclusion, dietary Zn-CS supplementation effectively enhances growth performance, antioxidant capacity, and endocrine function in weaned foals, while favorably modulating gut microbial composition. These findings underscore the potential of Zn-CS as a functional feed additive to support health and development during the critical weaning period, with 6 mg/kg body weight/day identified as the optimal supplementation level under the conditions of this study.

## Figures and Tables

**Table 1:** Composition and nutritional level of concentrate supplement (air dried material basis) %.

Item	Content
Mountain grass	72.00
Corn	12.88
Oats	4.20
Wheat bran	1.40
Soybean meal	7.56
Soybean oil	0.56
Premix ^(1)^	1.40
Total	100.00
Nutritional level ^(2)^	
DM	91.00
OM	94.28
CP	12.08
NDF	43.29
ADF	23.14
GE	15.04
Ca	0.75
P	0.42

^(1)^ The premix provided the following per kg of the concentrate supplement: VA 10 500 IU, VD3 2 200 IU, VE 15 IU, VK3 2 mg, VB1 1 mg, VB2 5 mg, VB6 3 mg, VB12 0.02 mg, D-biotin 0.10 mg, folic acid 1 mg, nicotinamide 20 mg, D-pantothenic acid 7 mg, Cu 12.5 mg, Fe 45 mg, Mn 39.4 mg. ^(2)^ Nutritional levels was measured. DM, dry matter; OM, organic matter; CP, crude protein; NDF, neutral detergent fiber; ADF, acid detergent fiber; GE, gross energy; Ca, calcium; P, phosphorus.

**Table 2:** Draw standard curve of VFA.

Number	1	2	3	4	5	6
Standard solution of mixed acid (mL)	0.05	0.1	0.2	0.3	0.4	0.5
ddH_2_O (mL)	0.45	0.4	0.3	0.2	0.1	0
4-Methylvaleric acid (internal standard, 40 mmol/L), added volume (mL)	0.1	0.1	0.1	0.1	0.1	0.1
10% TCA (mL)	0.5	0.5	0.5	0.5	0.5	0.5
Acetate (mmol/L)	6.9444	13.2252	30.3820	50.2204	66.6479	80.9264
Propionate (mmol/L)	5.7823	9.7711	20.3361	32.0894	42.2160	51.6452
Isobutyrate (mmol/L)	0.0509	0.2362	0.4750	0.7310	0.9699	1.1878
Butyrate (mmol/L)	3.7657	6.5741	13.0137	21.0230	27.1779	33.9058
Isovalerate (mmol/L)	0.2300	0.3575	0.6758	1.0933	1.4228	1.7583
Valerate (mmol/L)	0.0988	0.2699	0.6561	1.2006	1.5984	1.9920

**Table 3:** Effects of zinc cysteamine on apparent digestion and metabolism of nutrients in weaned foals (%).

Item	Control Group	2 mg/kg BW/d	4 mg/kg BW/d	6 mg/kg BW/d	SEM	*p*-Value		
						Treatment	Linear	Quadratic
Apparent digestibility (%)								
DM	51.35	53.81	55.54	57.43	2.94	0.223	0.040	0.891
OM	55.02	57.41	58.90	60.35	2.74	0.269	0.053	0.810
DE MJ/kg	6.71	7.02	7.84	7.80	0.59	0.166	0.037	0.687
CP	69.37	69.53	71.84	70.02	3.04	0.841	0.662	0.650
NDF	37.33	40.64	31.26	42.47	6.47	0.342	0.770	0.395
ADF	23.43	28.66	29.94	33.72	4.81	0.220	0.043	0.832
Ca	32.74	33.92	35.23	39.7	4.99	0.535	0.171	0.645
P	23.22	16.89	21.11	24.21	5.24	0.522	0.668	0.214
ME MJ/kg	6.51	6.79	7.62	7.61	0.59	0.164	0.036	0.730
Metabolic Rates of Nitrogen, Calcium and Phosphorus								
N	32.29	40.72	38.64	39.28	5.52	0.447	0.289	0.327
Ca	23.86	23.87	24.39	29.98	5.23	0.591	0.264	0.457
P	21.50	15.14	18.81	22.78	5.31	0.500	0.659	0.180

Abbreviations: DM, dry matter; OM, organic matter; DE, digestible energy; CP, crude protein; NDF, neutral detergent fiber; ADF, acid detergent fiber; Ca, calcium; P, phosphorus; ME, metabolizable energy; N, nitrogen. MJ/kg, megajoules per kilogram.

**Table 4:** Effects of zinc cysteamine on plasma biochemical indexes of weaned foals.

Item	Control Group	2 mg/kg BW/d	4 mg/kg BW/d	6 mg/kg BW/d	SEM	*p*-Value		
						Trt	Time	T × t
TP	62.71	63.39	63.81	62.02	0.56	0.120	<0.001	0.045
ALB	32.88 ^AB^	32.75 ^AB^	33.45 ^A^	32.02 ^B^	0.28	0.006	<0.001	0.529
GLB	29.77	30.64	30.36	29.64	0.55	0.52	<0.001	0.154
UREA	8.42	7.73	7.92	8.15	0.16	0.2576	<0.001	0.010
CREA-S	66.01 ^b^	75.31 ^a^	65.63 ^b^	65.53 ^b^	2.48	0.013	<0.001	0.172
Glu-G	5.22	5.06	5.56	5.38	0.17	0.197	<0.001	0.460
TC	2.02 ^B^	2.23 ^A^	2.07 ^AB^	2.07 ^AB^	0.04	0.004	<0.001	0.507
T-bil-D	11.14 ^b^	13.86 ^a^	13.58 ^a^	13.01 ^ab^	0.68	0.025	<0.001	0.513
AST	276.68 ^AB^	259.52 ^B^	286.12 ^A^	265.00 ^B^	5.84	0.008	<0.001	0.538
ALT	8.96 ^ab^	8.31 ^b^	9.72 ^a^	9.23 ^ab^	0.35	0.041	0.001	0.589
ALP	272.39 ^C^	278.55 ^BC^	324.76 ^A^	310.00 ^AB^	8.55	<0.001	<0.001	0.697
γ-GT	19.43	17.03	21.31	19.29	1.25	0.122	0.031	0.395
CK	308.08	262.09	262.35	245.51	21.18	0.190	<0.001	0.260
AST/ALT	33.62	32.35	30.58	30.47	1.64	0.470	0.010	0.912
Ca	2.72	2.73	2.71	2.72	0.02	0.943	<0.001	0.064
P	1.70	1.62	1.64	1.64	0.05	0.611	0.820	0.928
Mg	0.84 ^ab^	0.99 ^a^	0.77 ^b^	0.7 ^b^	0.07	0.025	0.056	0.079

Note: Values with different letters within the same row differ significantly. Values with different lowercase letters within the same row differ significantly at *p* < 0.05, whereas values with different uppercase letters differ significantly at *p* < 0.01. TP, total protein; ALB, albumin; GLB, globulin; UREA, urea; CREA-S, serum creatinine; Glu-G, glucose; TC, total cholesterol; T-bil-D, direct bilirubin; AST, aspartate aminotransferase; ALT, alanine aminotransferase; ALP, alkaline phosphatase; γ-GT, gamma-glutamyl transferase; CK, creatine kinase; Ca, calcium; P, phosphorus; Mg, magnesium.

**Table 5:** Effects of zinc cysteamine on plasma antioxidation of weaned foals.

Items	Control Group	2 mg/kg BW/d	4 mg/kg BW/d	6 mg/kg BW/d	SEM	*p*-Value		
						Trt	Time	T × t
SOD (U/mL)	75.935 ^Dd^	81.349 ^Cc^	88.220 ^Bb^	94.467 ^Aa^	1.249	<0.001	<0.001	0.254
GSH-Px (U/mL)	148.859 ^Dd^	162.078 ^Cc^	169.934 ^ABb^	179.015 ^Aa^	2.581	<0.001	<0.001	0.487
T-AOC (U/mL)	7.598 ^Dd^	8.664 ^Cc^	9.285 ^Bb^	10.070 ^Aa^	0.088	<0.001	<0.001	0.770
CAT (U/mL)	41.136 ^Dd^	45.957 ^BCc^	50.799 ^ABb^	55.408 ^Aa^	1.450	<0.001	<0.001	0.994
MDA (nmol/mL)	4.005 ^Dd^	3.587 ^BCc^	3.236 ^Bb^	2.970 ^Aa^	0.100	<0.001	<0.001	0.954

Note: Values with different letters within the same row differ significantly. Values with different lowercase letters within the same row differ significantly at *p* < 0.05, whereas values with different uppercase letters differ significantly at *p* < 0.01. SOD, superoxide dismutase; GSH-Px, glutathione peroxidase; T-AOC, total antioxidant capacity; CAT, catalase; MDA, malondialdehyde.

**Table 6:** Effects of zinc cysteamine on pH and VFA in feces of weaned foals.

Items	Control Group	2 mg/kg BW/d	4 mg/kg BW/d	6 mg/kg BW/d	SEM	*p*-Value		
						Treatment	Linear	Quadratic
pH	7.20 ^a^	6.77 ^b^	6.73 ^b^	6.65 ^b^	0.18	0.023	0.006	0.181
Acetate (µg/g)	2139.61	2268.84	2322.04	2652.22	352.68	0.523	0.165	0.690
Propionate (µg/g)	508.32	652.46	564.64	741.81	119.4	0.245	0.116	0.846
Butyrate (µg/g)	172.93	243.37	203.53	334.08	73.00	0.163	0.065	0.565
Isobutyrate (µg/g)	32.29	33.39	46.34	40.77	15.25	0.772	0.433	0.759
Valerate (µg/g)	29.87	32.76	43.29	66.76	17.30	0.157	0.035	0.407
Isovalerate (µg/g)	63.15	33.57	99.58	95.09	35.90	0.243	0.165	0.625
Acetate/Propionate	4.19	3.56	4.17	3.86	0.29	0.118	0.688	0.442
TVFA (µg/g)	2946.17	3264.39	3279.42	3930.73	582.79	0.405	0.118	0.689

Note: Values with different lowercase letters within the same row differ significantly at *p* < 0.05.

**Table 7:** Effects of zinc cysteamine on the diversity of fecal flora in weaned foals.

Items	Control Group	2 mg/kg BW/d	4 mg/kg BW/d	6 mg/kg BW/d	SEM	*p*-Value		
						Treatment	Linear	Quadratic
chao1	2094.20	2185.12	2047.97	2222.85	124.68	0.487	0.533	0.638
observed_features	2022.00	2116.63	1999.88	2161.5	118.18	0.482	0.426	0.692
dominance	0.02	0.01	0.01	0.01	0.01	0.588	0.297	0.677
shannon	9.08	9.49	9.14	9.57	0.34	0.398	0.310	0.977
pielou_e	0.83	0.86	0.83	0.86	0.03	0.465	0.322	0.942
goods_coverage	1.00	1.00	1.00	1.00	0.00	0.688	0.337	0.590
simpson	0.98	0.99	0.99	0.99	0.01	0.588	0.297	0.677

## Data Availability

The data that support the findings of this study are available from the corresponding author upon reasonable request.

## Supplementary Materials

The following supporting information can be downloaded at: https://www.mdpi.com/article/10.3390/ani16101568/s1, Table S1: Effects of Zn-CS supplementation on fecal bacterial phylum composition in weaned foals; Table S2: Effects of Zn-CS supplementation on fecal bacterial class level in weaned foals; Table S3: Effects of Zn-CS supplementation on fecal bacterial order level in weaned foals; Table S4: Effects of Zn-CS supplementation on fecal bacterial family level in weaned foals; Table S5: Effects of Zn-CS supplementation on fecal bacterial genus level in weaned foals; Table S6: Effects of Zn-CS supplementation on fecal bacterial species level in weaned foals; Table S7: Pairwise PERMANOVA (Adonis) results of beta-diversity differences among experimental groups based on Bray-Curtis distance.

## Abbreviations

The following abbreviations are used in this manuscript:

ADF	Acid detergent fiber
ALB	Albumin
ALP	Alkaline phosphatase
ANOVA	Analysis of Variance
BW	Body weight
Ca	Calcium
CAT	Catalase
CK	Creatine kinase
CP	Crude protein
CREA-S	Serum creatinine
CS	Cysteamine
DE	Digestible energy
DM	Dry matter
FID	Flame ionization detector
GC	Gas chromatograph
GE	Gross energy
GH	Growth hormone
Glu-G	Glucose
GLB	Globulin
GSH-Px	Glutathione peroxidase
I-Bil	Indirect bilirubin
INS	Insulin
LDA	Linear discriminant analysis
LDH	Lactate dehydrogenase
LEfSe	Linear discriminant analysis Effect Size
MDA	Malondialdehyde
ME	Metabolizable energy
Mg	Magnesium
N	Nitrogen
NDF	Neutral detergent fiber
OM	Organic matter
OTU	Operational taxonomic unit
P	Phosphorus
PCoA	Principal coordinates analysis
PCA	Principal component analysis
SCFA	Short-chain fatty acids
SEM	Standard Error of the Mean
SOD	Superoxide dismutase
SS	Somatostatin
T_3_	Triiodothyronine
T_4_	Thyroxine
T-AOC	Total antioxidant capacity
TC	Total cholesterol
T-Bil	Total bilirubin
TG	Triglycerides
TP	Total protein
TVFA	Total volatile fatty acids
UN	Urea nitrogen
VFA	Volatile Fatty Acids
Zn	Zinc
Zn-CS	Zinc Cysteamine
γ-GT	γ-glutamyl transferase
