# Supplementary material for: Effects of Dietary Zinc Cysteamine Supplementation on Growth Performance, Physiological Responses, and Fecal Microbiota in Weaned Foals
Source: Animals (Basel). 2026 May 21;16(10):1568. doi: 10.3390/ani16101568 (PMC13203130; doi:10.3390/ani16101568)
Supplement: Supplementary file 1 [file animals-16-01568-s001.zip › animals-4259493-supplementary.pdf]

Table S1. Effects of Zn-CS supplementation on fecal bacterial phylum composition in weaned foals.

Items	Control group	2 mg/kg BW/d	4 mg/kg BW/d	6 mg/kg BW/d
Firmicutes	66.73±13.85	71.76±2.63	69.23±6.44	65.1±5.35
Proteobacteria	5.62±15.67	0.11±0.07	0.10±0.04	0.13±0.07
Bacteroidota	17.97±5.05	18.52±5.92	18.65±8.27	24.05±9.39
Euryarchaeota	1.83±4.12	2.14±3.38	1.66±1.23	2.57±4.13
Verrucomicrobiota	4.64±3.03	4.72±3.34	6.30±3.36	4.83±2.86
Actinobacteriota	1.12±1.49	0.80±0.38	0.86±0.60	0.90±0.98
Patescibacteria	1.14±1.06	1.11±0.52	2.16±1.02	1.46±1.37
Spirochaetota	0.51±0.40	0.50±0.47	0.57±0.33	0.50±0.52
Synergistota	0.11±0.17	0.03±0.02	0.06±0.04	0.06±0.07
Cyanobacteria	0.05±0.04	0.06±0.04	0.07±0.02	0.15±0.14
Others	0.27±0.11	0.24±0.11	0.33±0.18	0.26±0.10

Table S2. Effects of Zn-CS supplementation on fecal bacterial class level in weaned foals.

Items	Control group	2 mg/kg BW/d	4 mg/kg BW/d	6 mg/kg BW/d
Clostridia	61.42±14.41	63.84±5.07	58.77±8.08	57.69±6.06
Gammaproteobacteria	5.59±15.67	0.08±0.08	0.06±0.04	0.1±0.06
Bacteroidia	17.97±5.05	18.52±5.92	18.65±8.27	24.05±9.39
Bacilli	4.24±1.76	6.75±5.83	9.1±7.09	5.92±3.8
Methanobacteria	1.83±4.12	2.14±3.38	1.66±1.23	2.57±4.13
Verrucomicrobiae	3.76±2.63	3.64±2.82	5.34±2.78	2.79±2.07
Kiritimatiellae	0.88±0.87	1.08±1.25	0.96±0.87	2.04±2.4
Actinobacteria	0.59±1.56	0.10±0.05	0.08±0.04	0.10±0.15
Saccharimonadia	1.07±1.08	1.07±0.53	2.07±0.93	1.44±1.38
Negativicutes	0.83±0.43	0.91±0.53	1.09±0.58	1.19±1.02
Others	1.82±0.66	1.88±0.51	2.22±0.73	2.12±0.90

Table S3. Effects of Zn-CS supplementation on fecal bacterial order level in weaned foals.

Items	Control group	2 mg/kg BW/d	4 mg/kg BW/d	6 mg/kg BW/d
Pseudomonadales	5.55±15.68	0.03±0.05	0.00±0.00	0.05±0.08
Oscillospirales	24.04±6.68	25.56±6.31	24.45±5.92	26.11±6.95
Lachnospirales	23.65±9.10	23.94±2.91	20.08±5.74	19.11±4.82

Bacteroidales	17.93±5.03	18.47±5.90	18.62±8.28	23.98±9.36
Lactobacillales	3.05±1.75	5.53±5.51	7.48±6.38	4.38±3.42
Methanobacteriales	1.83±4.12	2.14±3.38	1.66±1.23	2.57±4.13
Christensenellales	6.15±2.69	6.21±1.51	6.29±1.11	5.71±2.17
Verrucomicrobiales	3.75±2.63	3.64±2.82	5.33±2.78	2.79±2.07
WCHB1-41	0.88±0.87	1.08±1.25	0.96±0.87	2.04±2.40
Peptostreptococcales-Tissierellales	3.59±1.31	3.49±0.83	3.12±0.93	2.97±1.60
Others	9.59±2.45	9.92±1.62	12.00±3.10	10.3±2.67

Table S4. Effects of Zn-CS supplementation on fecal bacterial family level in weaned foals.

Items	Control group	2 mg/kg BW/d	4 mg/kg BW/d	6 mg/kg BW/d
Moraxellaceae	5.54±15.68	0.03±0.05	0.00±0.00	0.05±0.08
Lachnospiraceae	23.37±9.00	23.63±2.90	19.73±5.64	18.67±4.58
Oscillospiraceae	13.76±3.73	14.44±3.83	14.99±4.64	15.57±5.36
Streptococcaceae	2.26±1.59	4.74±5.09	6.83±6.12	3.75±3.33
Prevotellaceae	6.73±3.60	6.19±2.12	7.75±3.80	8.45±4.67
F082	3.43±2.41	4.34±2.30	3.94±2.71	5.28±3.29
Methanobacteriaceae	1.83±4.12	2.14±3.38	1.66±1.23	2.57±4.13
Christensenellaceae	6.15±2.69	6.21±1.51	6.29±1.11	5.71±2.17
Rikenellaceae	4.08±1.62^{ab}	4.62±1.69^{ab}	3.20±1.06^{b}	5.60±3.01^{a}
Akkermansiaceae	3.75±2.63	3.64±2.82	5.33±2.78	2.79±2.07
Others	29.10±3.79	30.03±3.47	30.27±3.19	31.57±4.71

Table S5. Effects of Zn-CS supplementation on fecal bacterial genus level in weaned foals.

Items	Control group	2 mg/kg BW/d	4 mg/kg BW/d	6 mg/kg BW/d
Psychrobacter	5.54±15.68	0.03±0.05	0.00±0.00	0.05±0.08
Streptococcus	2.26±1.59	4.74±5.09	6.83±6.12	3.75±3.33
Methanobrevibacter	1.83±4.12	2.14±3.38	1.66±1.23	2.57±4.13
Prevotellaceae_UCG-001	3.82±2.68	2.96±1.19	4.39±2.84	3.94±3.34
Christensenellaceae_R-7_group	5.81±2.50	5.86±1.47	6.01±1.06	5.36±2.11
NK4A214_group	5.58±1.22	5.53±0.92	6.37±2.16	5.91±2.12
Akkermansia	3.75±2.63	3.64±2.82	5.33±2.78	2.79±2.07
_Rikenellaceae_RC9_gut_group	3.83±1.55^{ab}	4.29±1.53^{ab}	2.99±0.98^{b}	5.27±2.87^{a}

Lachnospiraceae_AC2044_group	3.26±2.32	2.67±1.24	1.94±1.37	2.39±1.54
UCG-002	3.18±1.32	3.19±1.77	3.34±1.86	3.47±2.20
Others	61.12±9.71	64.95±6.26	61.14±5.78	64.5±7.09

Table S6. Effects of Zn-CS supplementation on fecal bacterial species level in weaned foals.

Items	Control group	2 mg/kg BW/d	4 mg/kg BW/d	6 mg/kg BW/d
Methanobrevibacter_ruminantium	0.37±0.90	0.28±0.45	0.17±0.17	0.33±0.40
Clostridium_butyricum	0.44±0.61	0.41±0.67	0.35±0.26	0.22±0.24
Lactobacillus_hayakitensis	0.27±0.18	0.33±0.22	0.31±0.34	0.27±0.20
Lactobacillus_equi	0.22±0.11	0.28±0.23	0.24±0.26	0.23±0.17
rumen_bacterium_NK4A65	0.29±0.22^{ab}	0.32±0.11ab	0.36±0.17^{a}	0.18±0.13^{b}
Ruminococcus_sp_YE281	0.01±0.00	0.01±0.02	0.03±0.03	0.09±0.23
Ruminococcus_flavefaciens	0.21±0.21	0.12±0.09	0.07±0.07	0.12±0.14
Bacteroidales_bacterium_Bact_22	0.01±0.01	0.05±0.15	0.08±0.18	0.07±0.20
Clostridiales_bacterium_Firm_14	0.28±0.10^{ab}	0.24±0.13ab	0.36±0.10^{a}	0.20±0.14b
Ruminococcus_sp_HUN007	0.13±0.11	0.16±0.14	0.10±0.09	0.17±0.16

Table S7. Pairwise PERMANOVA (Adonis) results of beta-diversity differences among experimental groups based on Bray-Curtis distance.

Group	Df	SumsOfSqs	MeanSqs	F.Model	R2	Pr(>F)
Control group-2 mg/kg BW/d	1(14)	0.16868(2.75165)	0.16868(0.19655)	0.8582	0.05776(0.94224)	0.754
Control group-4 mg/kg BW/d	1(14)	0.18698(2.8474)	0.18698(0.20339)	0.91932	0.06162(0.93838)	0.565
Control group-6 mg/kg BW/d	1(14)	0.23508(2.98894)	0.23508(0.2135)	1.10108	0.07291(0.92709)	0.26
2 mg/kg BW/d-4 mg/kg BW/d	1(14)	0.1659(2.57351)	0.1659(0.18382)	0.90249	0.06056(0.93944)	0.592
2 mg/kg BW/d-6 mg/kg BW/d	1(14)	0.15904(2.71505)	0.15904(0.19393)	0.8201	0.05534(0.94466)	0.735
4 mg/kg BW/d-6 mg/kg BW/d	1(14)	0.22337(2.8108)	0.22337(0.20077)	1.11254	0.07362(0.92638)	0.303
